# Statement of Retraction: Identification of a novel circular RNA circZNF652/miR-486-5p/SERPINE1 signaling cascade that regulates cancer aggressiveness in glioblastoma (GBM)

**DOI:** 10.1080/21655979.2025.2491956

**Published:** 2025-04-23

We, the Editors and Publisher of the journal *Bioengineered*, have retracted the following article:

Liu, L., Xiao, S., Wang, Y., Zhu, Z., Cao, Y., Yang, S., … Zheng, Y. (2022). Identification of a novel circular RNA circZNF652/miR-486-5p/SERPINE1 signaling cascade that regulates cancer aggressiveness in glioblastoma (GBM). *Bioengineered, 13*(1), 1411–1423. https://doi.org/10.1080/21655979.2021.2018096

Since publication, significant concerns have been raised about the integrity of the data and reported results in the article. When approached for an explanation, the authors did not provide their original data or any necessary supporting information. As verifying the validity of published work is core to the integrity of the scholarly record, we are therefore retracting the article. The corresponding author listed in this publication has been informed.

We have been informed in our decision-making by our editorial policies and the COPE guidelines.

The retracted article will remain online to maintain the scholarly record, but it will be digitally watermarked on each page as ‘Retracted’.

